# Climate change and increased risk of respiratory infections in humans 

**DOI:** 10.2471/BLT.25.294800

**Published:** 2025-10-01

**Authors:** Nicola Abrescia, Maurizio D’Abbraccio, Adelaide Maddaloni, Gabriella Molinaro

**Affiliations:** a Azienda Ospedaliera di Rilievo Nazionale dei Colli, D. Cotugno Hospital for Infectious Diseases, Via Gaetano Quagliariello 54, 80131, Naples, Italy.; b Department of Clinical Medicine and Surgery, University Federico II of Naples, Italy.

Climate change has become an immediate crisis with profound implications for global health.^1^ Rising temperatures, altered precipitation patterns, worsening air pollution and extreme weather events are now strongly associated with marked changes in infectious disease dynamics.^2^ These climatic shifts have expanded the range of vectors, increased the prevalence of waterborne infections and disrupted ecosystems.^3^^,^^4^ Such environmental transformations also create ideal conditions for the proliferation and transmission of respiratory pathogens, thereby intensifying the incidence and severity of respiratory infections.^5^

The international health community increasingly recognizes the relationship between climate change and infectious diseases, yet the specific threat to respiratory health remains underappreciated. Alterations in temperature and humidity influence the stability, survival and dispersion of airborne pathogens.^5^ Elevated temperatures also encourage fungal proliferation, increasing exposure to airborne spores. The incidence of coccidioidomycosis has risen sharply in the southwestern United States of America, with climate-driven drought identified as a key factor.^6^ These changes, compounded by deteriorating air quality, heighten population exposure to respiratory pathogens, particularly in regions undergoing rapid environmental degradation.^7^^,^^8^

Air pollution is a critical driver linking climate change and respiratory infections.^5^ Evidence from a European pooled cohort study, including 325 367 participants and 712 pneumonia- and influenza-related deaths, showed that long-term exposure to nitrogen dioxide (NO_2_) and black carbon was associated with a 10–12% increase in mortality from these infections.^9^ Wildfire smoke, an increasingly frequent consequence of climate change, has also been linked to surges in respiratory infections, especially among vulnerable populations.^8^ Climate change exacerbates air pollution through the accumulation of greenhouse gases and fine particulate matter (PM_2.5_ and PM_10, _respectively), both of which directly impair respiratory health.^10^ NO_2_, primarily from fossil fuel combustion, accumulates under stagnant atmospheric conditions,^10^ while elevated temperatures accelerate photochemical reactions, increasing ground-level ozone, a well-recognized respiratory irritant.^10^ Wildfires further elevate PM_2.5_ and NO_2_ levels,^8^ and dust storms contribute to PM_10_ levels, aggravating respiratory morbidity.^10^

Gaseous pollutants can undergo chemical reactions in the atmosphere to form secondary particulate matter. These particles, particularly PM_2.5_, penetrate deeply into the lungs and cause greater harm than their gaseous precursors by inducing oxidative stress, inflammation and impaired immune defence. The formation of secondary PM is accelerated by high temperatures and increased solar radiation, linking climate change directly to worsened respiratory risks.^10^ Another important phenomenon is the urban heat island effect, whereby heat-absorbing surfaces such as asphalt and concrete cause elevated urban temperatures, increasing secondary PM_2.5_ formation during heatwaves. Urban emissions from vehicles and industry, combined with this effect, further raise PM concentrations. In addition, agricultural burning in climate-affected regions increases PM_2.5_ levels, worsening air quality and respiratory health outcomes.^10^

Air pollutants cause direct injury to the respiratory system. Prolonged exposure to PM, ozone, NO_2_ and sulfur dioxide disrupts epithelial integrity, compromising the lungs’ first line of defence.^11^ Fine particles penetrate deeply into the alveoli, impairing gas exchange and triggering chronic inflammatory responses.^11^ Long-term inflammatory processes can progress to interstitial fibrosis, resulting in stiffened lung tissue and reduced elasticity.^11^

Air pollutant exposure also suppresses immune function,^12^ while upregulating viral entry receptors, such as the receptor to which Severe Acute Respiratory Syndrome Coronavirus 2 (SARS CoV-2) binds, potentially facilitating more severe viral infections.^12^ The combination of chronic inflammation, fibrosis and immune suppression in the respiratory tract fosters conditions favourable to the colonization of airborne pathogens.^12^ Respiratory viruses may invade deeper pulmonary structures^12^ that are already compromised by pollutant-induced inflammation and fibrosis. Pre-existing oedema, inflammation and fibrosis can exacerbate viral pneumonia and further impair gas exchange, leading to respiratory failure. This pathophysiological cascade may partly explain the high incidence and severity of pneumonia among patients with coronavirus disease 2019 (COVID-19) residing in polluted areas during the early pandemic,^9^ although factors such as population density, health-care capacity and underlying health conditions also likely contributed.

Through its impact on air pollution and pathogen transmission dynamics, climate change is creating a convergence of risk factors, an ideal environment for respiratory infections. Elevated pollutant exposure, weakened respiratory defences and shifting transmission patterns are together driving a substantial increase in the global burden and severity of respiratory diseases.^5^^,^^7^

Addressing the growing impact of climate change on the global burden of respiratory infections requires a coordinated response spanning environmental policy, clinical practice and public health action. The World Health Organization’s *Air Quality Guidelines*^10^ and the United Nations Environment Programme^1^ both call for urgent, integrated measures to mitigate these risks. Without such intervention, the surge of respiratory infections may become one of the gravest public health consequences of the climate crisis.
